# Adverse outcomes associated with the treatment of *Toxoplasma* infections

**DOI:** 10.1038/s41598-020-80569-7

**Published:** 2021-01-13

**Authors:** Ahmed M. Shammaa, Thomas G. Powell, Imaan Benmerzouga

**Affiliations:** Department of Biomedical Sciences, West Virginia School of Osteopathic Medicine, Lewisburg, WV USA

**Keywords:** Infectious diseases, Parasitic infection, Adverse effects, Antimicrobial therapy

## Abstract

Adverse outcomes associated with the treatment of *Toxoplasma gondii* infections in patients with various health backgrounds have not been characterized. The aim of this study was to identify the adverse outcomes and adverse events associated with the current clinical treatments of *Toxoplama gondii* infections using real world data reported to the FDA adverse event reporting system (FAERS). Data submitted to FAERS between 2013 and 2019 was retrieved and analyzed. Reporting odds ratio of death was calculated for the drugs having ≥ 25 reports of adverse outcomes. The adverse event profiles for the same drugs were analyzed and the reporting odds ratio was calculated relative to all other drugs used in the treatment of *Toxoplasma* infections. There were 503 cases reporting the treatment of *Toxoplasma* infections in the FAERS database. Death (DE) was the adverse outcome in 102 reports, of which 23 (22.5%) anti-*Toxoplasma* drugs were listed as the primary suspect drug (PS). Clindamycin (2.04; 1.07–3.90) followed by pyrimethamine (1.53; 0.99–2.36) were the most likely to be associated with death. Adverse events analysis suggest that sulfonamides formulations may have a less favorable safety profile. Our study represents the first real-world analysis of adverse outcomes and events associated with the treatment of *Toxoplasma* infections. Our findings support the need to better understand the current first-line agents for *Toxoplasma* infections, in addition to underscoring the need to identify safer regimens.

## Introduction

*Toxoplasma* gondii is a protozoan parasite with a variety of intermediate hosts including humans^1^. It is estimated that *Toxoplasma* has infected 30%-50% of the world’s population^2^. *Toxoplasma* utilizes its host machinery factors to replicate and divide and as a result can cause severe disease in the immunocompromised and congenital infected newborns^3,4^. The parasite has the ability to persist for the life-time of its host^5^, making its ubiquitous nature a threat to prone populations battling with weakened immune system. The golden standard for the clinical treatment of *Toxoplasma* infections includes a combination of two antimicrobial agents that target the folate pathway^6^. These treatments have known adverse event profiles^7–10^, but with little room for alternatives, they continue to be the default treatment for *Toxoplasma* infections^8^.

The FDA adverse events reporting system (FAERS) is a database where healthcare providers, pharmaceutical companies and consumers submit adverse events and medication error reports^11,12^. The database contains de-identified patient information in cases organized into tables containing reports on the regimens, indications, adverse events and outcomes of the used regimens^13^. Therefore, we aimed to describe the adverse outcomes and events for the clinically used treatments for *Toxoplasma* infections. We hypothesized that the current first line treatments will differ in their safety profiles in the publically available spontaneous reporting system and that these treatments will share common co-occurring diseases with *Toxoplasma* infection manifestations.

## Results

### *Toxoplasma* infections and treatments in FAERS

**Figure 1:**
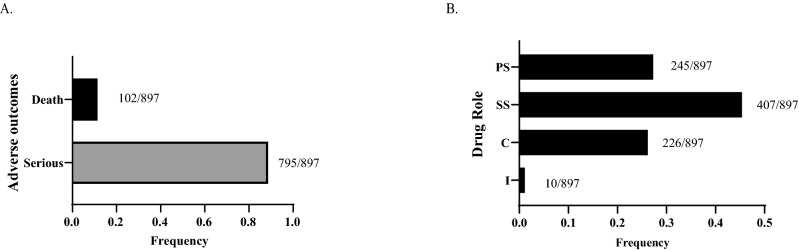
Summary of adverse outcomes in the treatment of *Toxoplasma* infections in FAERS database. (**A**) Summary of adverse outcomes from the cases treating *Toxoplasma* infections. (**B**) Specification of the role of the drug in the adverse outcome as listed in FAERS database.

From 2013–2019, there were a total of 503 cases treating *Toxoplasma* infections identified in the FAERS database. All of these cases included complete reports of indications, drugs, adverse events and outcomes. The baseline characteristics reported for the cases are summarized in Table 1. The mean age of patients was 46.5 years (SD 17). A total of 897 drugs treating *Toxoplasma* infections were identified from the cases. The majority of the adverse outcome reports were serious (795 [89%] of 897 adverse outcomes), followed by the adverse outcome of death (102 [11%] of adverse outcomes; Fig. 1A). The role of the anti-*Toxoplasma* agents were analyzed based on their role in the adverse outcome with the most frequent drug role being the secondary suspect drug (407 [45%] of 897 drugs), followed by the primary suspect drug (245 [27%] of drug; Fig. 1B).

**Table 1:** Baseline information.

	Toxoplasma infections (%)
Number of cases	503
**Age**	
0–1	6 (1)
> 1–20	21 (4)
> 20–60	301 (60)
> 60	89 (18)
Unknown	86 (17)
**Sex**	
Female	245 (46)
Male	233 (49)
Unknown	25 (5)
**Reporter country**	
US	193 (38)
France	142 (28)
Other countries	154 (31)
Unknown	14 (3)
**Reporter occupation**	
Physician	121 (24)
Pharmacist	92 (18)
Other health-professional	204 (41)
Consumer	77 (15)
Unknown	9 (2)

### Adverse outcomes associated with the treatment of *Toxoplasma* infections

**Figure 2:**
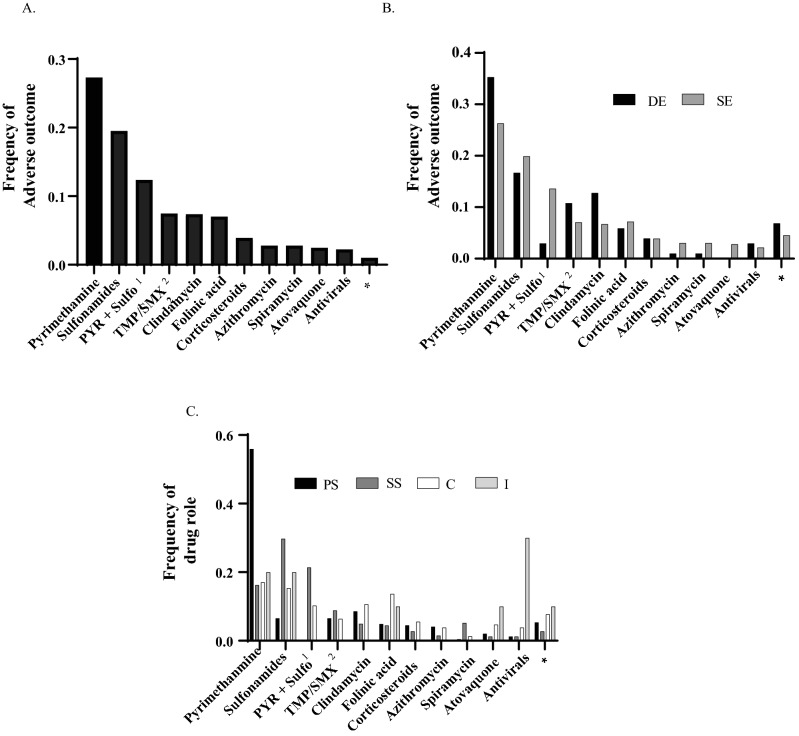
Drugs used in the treatment of *Toxoplasma* infections in FAERS database. (**A**) Drugs with > 10 adverse outcome reports. (**B**) Frequency of adverse outcome by agent/drug. (**C**) Role of the drug in the adverse outcome. (*) See table S4 for a list of drugs with < 10 adverse outcome reports. ^1^pyrimethamine, ^2^trimethoprim-sulfamethaxazole, Death (DE), Serious (SE).

The agents reported to be used with the indication of a *Toxoplasma* infection were analyzed for their frequency of causing an adverse outcome. The majority of adverse outcome reports were caused by pyrimethamine (245 [27%] of 897 adverse outcomes), followed by sulfonamides containing drugs (175 [20%] of adverse outcomes) and formulations that combined both pyrimethamine and sulfonamides (111 [12%] of adverse outcomes; Fig. 2A). The same set of drugs were analyzed for their frequency in the adverse outcome of death or serious adverse outcomes. The majority of the reports associated with death were associated with pyrimethamine (36 [35%] of 102 death outcome reports) followed by sulfonamides (17 [17%] of death outcome reports) and clindamycin (13 [13%] of death outcome reports; Fig. 2B). Additionally, the majority of the serious reports were associated with pyrimethamine (209 [26%] of 795 serious outcome reports), followed by sulfonamides (158 [20%] of 795 serious outcome reports) and the combination of pyrimethamine and sulfonamides (108 [13.5] of 795 serious outcome reports; Fig. 2B). The same set of drugs were further analyzed for their role in the adverse outcome with pyrimethamine (137 [56%] of 245 PS drug reports) followed by clindamycin (21 [9%] of PS drug reports) having the most adverse outcome reports as a primary suspect drug. Sulfonamide drugs (121 [30%] of 407 SS drug reports) followed by pyrimethamine plus sulfonamide (87 [21%] of SS drug reports) were the most secondary suspect drugs in the adverse outcome reports. Pyrimethamine (40 [17%] of 235 C drug reports) followed by sulfonamides (36 [15%] of C drug reports) were the most concomitant drugs in the adverse outcome reports. Finally, pyrimethamine or sulfonamide (2 [20%] of 10 I drug reports; Fig. 2C) were the most interacting drug in the adverse outcome reports.

**Figure 3:**
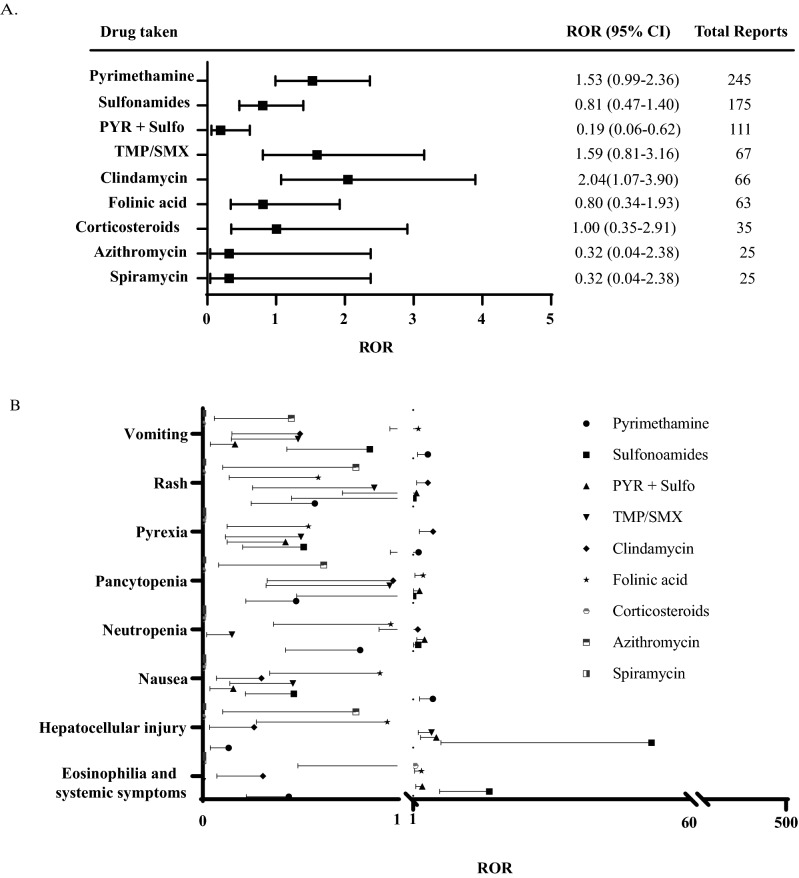
Reporting odds ratio (ROR) for the drugs with the most adverse outcome reports in FAERS database. (**A**) ROR of the adverse outcome of death for the drugs with the most adverse outcome reports. (**B**) ROR of the most common adverse events among the drugs used in the treatment of *Toxoplasma* infection in FAERS database.

We calculated the reporting odds ratio (95% CI) for the adverse outcome of death associated with the anti-*Toxoplasma* agents with ≥ 25 [3%] adverse outcome reports. A signal was detected for the adverse outcome of death with clindamycin only (2.04: 1.07–3.90: p = 0.0298; Fig. 3A). Pyrimethamine (1.53; 0.99–2.36: p = 0.056) was the second most likely drug with a signal for the outcome of death followed by TMP/SMX (1.59; 0.81–3.16: p = 0.1796). However, the association was not significant for pyrimethamine or TMP/SMX. We calculated the reporting odds ratio for the outcome of death for all indications in the database excluding the *Toxoplasma* infections for clindamycin (0.711; 0.68–0.75), pyrimethamine (2.10; 1.58–2.79) and TMP/SMX (1.73; 1.69–1.77) (Fig S2A).The adverse event profile was analyzed and we calculated the reporting odds ratio relative to all other drugs used in the treatment of *Toxoplasma* infections in the FAERS database. Only the most common adverse event reports were analyzed. The highest ROR was for hepatocellular injury (51.97; 6.96–387.77) associated with sulfonamides followed by drug reaction with eosinophilia and systemic symptoms (17.32: 6.67–45.01) associated with sulfonamides and nausea (5.26; 2.32–11.91; Fig. 3B) associated with pyrimethamine. The same set of adverse events were calculated for all other indications in the FAERS database using clindamycin, pyrimethamine or TMP/SMX and the highest ROR was for drug reaction with eosinophilia and systemic symptoms (51.86; 33.94–79.24) associated with pyrimethamine followed by pancytopenia (35.67; 24.06–52.88) associated with pyrimehtanmine and neutropenia (24.44; 17.50–34.14; Fig. S2B) associated with pyrimethamine.

A subsequent analysis was carried out for clindamycin, pyrimethamine and TMP/SMX. For clindamycin, the cases with the reported adverse outcome of death were for the treatment of cerebral toxoplasmosis (2 [15%] out of 13 cases) or toxoplasmosis (11 [85%] of 13 cases; Fig. S1B). The majority of the cases (10 [70%] out of 13 cases; Fig. S1A) were cases listing clindamycin as a concomitant drug. All but one case lists pyrimethamine as one of the agents used in the treatment of the *Toxoplasma* infection. Like clindamycin, the cases associated with the outcome of death when pyrimethamine was used were for cerebral toxoplasmosis (6 [17%] out of 36 cases) or toxoplasmosis (30 [83%] out of 36 cases; Fig. S1B). The majority of the cases (17 [48%] out of 36 cases) were cases listing pyrimethamine as a concomitant drug followed by (13 [36%] out of 36 cases; Fig. S1A) listing pyrimethamine as a primary suspect drug. All but one of the cases listing pyrimethamine as a primary suspect drug did not list any other anti-*Toxoplasm*a agent. For TMP/SMX the cases with the reported adverse outcome of death were for the treatment of cerebral toxoplasmosis (4 [36%] out of 11 cases) or toxoplasmosis (7 [64%] of 11 cases; Fig. S1B). The majority of the cases (7 [64%] out of 11 cases) were cases listing TMP/SMX as a secondary suspect drug followed by (3 [27%] out of 11 cases; Fig. S1A) listing TMP/SMX as primary suspect drug. All but one case listing TMP/SMX as a primary suspect drug did not list any other anti-*Toxoplasma* agent.

### Co-occurring diseases and *Toxoplasma* infections in FAERS

Finally, we analyzed the diseases co-occurring with *Toxoplasma* infections cases in the FAERS database. The diseases were grouped based on disease group or organ system. The diseases with the highest frequency of occurrence with *Toxoplasma* infection reports in FAERS include HIV/AIDS (550 [32%] of 1719 reported indications), followed by infectious diseases (472 [23%] of 1719 reported indication) and cancer (147 [9%] of 1719 reported; Table 2).

**Table 2:** Frequency of co-occurring diseases with *Toxoplasma* infections in FAERS database.

Disease	Total Reports (n = 1719)
HIV/AIDS	550 (32%)
Infectious diseases	472 (23%)
Cancer	147 (9%)
Neurological Disorders	110 (6%)
CV/Renal	78 (5%)
Inflammation and Immune Disorders	78 (5%)
Immunomodulation and Transplant	65 (4%)
Psychiatric Disorders	62 (4%)
Eye related conditions	32 (2%)
Endocrine	31 (2%)
Other	27 (2%)
Blood Disorders	23 (1%)
Gastrointestinal	22 (1%)
Respiratory	16 (1%)
Reproductive	5 (< 1%)

## Discussion

In this spontaneous reporting system study using the FAERS database from 2013–2019, death was reported in 102 (11%) of 897 adverse outcomes associated with the treatment of *Toxoplasma* infections. The regimens utilized for *Toxoplasma* infections were mostly secondary suspect drugs in their adverse outcomes, pyrimethamine or sulfonamides were the drugs with the most adverse event reports, a safety signal for the outcome of death was detected with the use of clindamycin and the most frequent co-occurring disease with *Toxoplasma* infections was HIV/AIDS.

It is estimated that approximately 30% of humans are infected by *Toxoplasma*, causing life-threatening disease in the immunocompromised^4^. While prevention measures are primarily focused on prevention of infection and screening susceptible population for *Toxoplasma* infection^4^, improved understanding of the available treatments and the development of alternative treatments remains an on-going effort with significant challenges^8^. A strength of this study is the evaluation of the adverse outcome and event profiles of current clinical treatments of *Toxoplasma* infections in a database that provides co-occurring treatments and diseases, allowing us to have a better understanding of the anti-*Toxoplasma* agents in a clinical setting.

We have observed that among all of the reports of adverse events submitted to the FDA, pyrimethamine, sulfonamides or their combination had the most adverse event reports submitted to the database. The golden standard for the treatment of *Toxoplasma* infections remains a combination therapy of agents that target the folate pathway despite significant adverse events that have been reported^6,14^. Although this study is limited by the limitation of the database^15^, we observed a safety signal for the adverse outcome of death with clindamycin with the indication of *Toxoplasma* infections. This signal was not observed when the database was searched for the use of clindamycin with all other indications. The efficacy of antibiotic treatment against *Toxoplasma* infections is poorly studied^16^. However, subsequent analysis revealed that the cases associated with the adverse outcome of death with clindamycin included pyrimethamine in the drug file. Therefore, it is likely that these reports may present disease progression, dosing error or treatment failure, as regimens for *Toxoplasma* infections with clindamycin have been shown to be less effective^14^. The reporting odds ratio for the adverse outcome of death was not significant for *Toxoplasma* infections indications but significant for all other indications in the database that included pyrimethamine or TMP/SMX supporting previous findings that these agents can have life-threatening adverse events^7,17^. Finally, the highest reporting odds ratio for the analyzed adverse events was for sulfonamides, suggesting an increased likelihood of adverse event reporting^18^. This observation is consistent with the clinical observations of sulfonamides hypersensitivity in the treatment of *Toxoplasma* infections^19^ necessitating alternative treatments^14,20^ as more adverse events are likely to be reported with sulfonamides.

Pyrimethamine is one of the best studied and characterized anti-*Toxoplasma* agent relative to other drugs^7^. However, due to the toxicity of pyrimethamine, factors that influence the success of the treatment of *Toxoplamsa* infections have not been well characterized as alternative therapies are indicated. In particular, toxoplasmosis is a significant burden to immunocompromised^21^ individuals and to infants via maternal transmission^22^. While several reports exist that evaluate the interaction of *Toxoplasma* with co-occurring diseases^23–25^, it remains unclear as to how does *Toxoplasma* infection impacts progression of the common co-occurring diseases receiving treatments in a clinical setting and whether treatment failure or disease progression or other causes leads to the observed associations. There are few studies that evaluate the efficacy of anti-*Toxoplasma* agents in the presence of other treatments^26^. There are also reports of the possibility of anti-*Toxoplasma* activity in other drug classes^27^. Therefore, a better understanding of the interplay between treatments, *Toxoplasma* infection and co-occurring diseases is needed.

This study has limitations that must be addressed. First we used a spontaneous adverse event reporting system. Therefore, like many other databases the FAERS suffers from both common and unique limitations to a spontaneous reporting system^15,28^. However, we ensured that the data analyzed contained complete reports for the indications, treatments, adverse events and outcomes. Second, we used reporting odds ratio as a method to detect an association with an outcome or event^29^. The association between an adverse event or outcome and a drug is best assessed by a randomized, prospective, large scale and long-term clinical trial. However, the heterogeneous nature of *Toxoplasma* infections^30^, the different patient backgrounds and co-occurring diseases and treatment limitation due to patient intolerance^14^ create a restrictive limitation on conducting such studies. Therefore, data mining of the spontaneous adverse event reporting system might reveal clinically important information and FAERS database has already been shown to reproduce already established clinical associations^11^. Third, the database relies on voluntary reporting by healthcare professionals and consumers^15^, which can be influenced by a variety of factors including healthcare professional’s attitude towards reporting^31^. Forth, the designation of the drug role is subjective and other factors may influence the adverse outcome and cannot be ruled out using this database^32^. Finally, the database does not require evidence or details for the reported cases^15^ and does not provide drug usage for rate calculation^32^.

Our study is the first study that provides a detailed analysis of anti-*Toxoplasma* agents and their association with an adverse outcome and various adverse events in the FAERS database showing that a better understanding of these agents when used clinically is needed. Despite the limitation of the database, our data supports previous findings on the toxicity of current anti-*Toxoplasma* agents from real-world data, thus underscoring the need to develop or identify strategies to improving current treatments.

## Methods

### Study design

FAERS quarterly data extracts from 2013 until 2019 were downloaded from the download page on the FDA website (https://fis.fda.gov/extensions/FPD-QDE-FAERS/FPD-QDE-FAERS.html). The database was searched for reports of adverse outcomes in the outcomes file (OUTC) associated with the treatment of a *Toxoplasma* infection (Table S1) in the indication file (INDI). Duplicate reports were identified and eliminated by keeping the most recent case version as recommended by the FDA^18^. Available demographic information was obtained for the cases, including age, sex, reporter country and occupation using the demographics file (DEMO). The outcomes in the FAERS are coded as one or more of seven types of outcomes given in the FAERS information file “ASC_NTS.doc”. The outcomes were re-categorized as follows: Death (DE) was assigned to cases which include an outcome of death and serious (SE) was assigned to cases with all other outcomes listed in the FAERS information file (Table S2). The frequency of each category was assessed in *Toxoplasma* infection cases. Cases were cross-referenced with the drug (DRUG) file, which contains the name of each drug as well as the role of the drug in the development of the adverse event. The frequency of the role of the drug in the adverse event was assessed in terms of the FAERS categories of primary suspect (PS), secondary suspect (SS), concomitant (C), and interacting (I). Since death (DE) was the most serious adverse outcome reported, we calculated the reporting odds ratio (ROR) for death (DE) for each of the drugs associated with ≥ 25 of adverse outcome reports. We also calculated the ROR for the most common adverse drug events (ADEs) reported for these drugs by cross-referencing with the reaction file (REAC). The same approach was used for the extraction of data for all other indications other than *Toxoplasma* infections for clindamycin, pyrimethamine and TMP/SMX. Finally, we determined the frequencies of all indications that were co-occurring with the *Toxoplasma* infections cases.

### Data extraction

To facilitate data extraction and analysis, a Python script was developed. Each case in the database is identified by a unique "primary ID". The “primary ID” was used to compile information for a given case from the 7 different tables in the database. The program first identified all cases from 2013 to 2019 with a value in the "indi_pt" field matching any of the following terms: "Cerebral toxoplasmosis", "Toxoplasmosis", "Congenital toxoplasmosis", or "Eye infection toxoplasmal". These terms were identified from searching the FAERS database for *Toxoplasma* manifestations. The resulting list of primary IDs was then used to extract selected fields from the DEMO, DRUG, REAC, OUTC, and INDI data tables for each year. Finally, these data were merged together into one dataset and copied to excel for statistical analysis.

Drug names in the FAERS database are reported as brand names, generic names, or abbreviations and can have spelling inconsistencies^33^. A text-mining approach was used to group together drugs based on their chemical formulation. Agents with fewer adverse outcome reports were grouped together into broader categories (Table S3).

### Statistical analysis

Reporting odds ratio (ROR) was calculated as described previously^18^. A signal was defined when the lower limit of the 95% CI > 1. Descriptive statistics was used to obtain the results. Frequencies and percentages were used for categorical variables. Mean and standard deviation were used for the reported age. Data was analyzed using excel 2016 and Graphpad prism version 8.0.

### Ethical approval

The WVSOM Institutional Review Board reviewed the proposal and provided the following statement “The WVSOM Institutional Review Board has reviewed the non-human subjects research determination request form and email communications for the above-identified research. OHRP does not consider research involving publicly available, de-identified data to involve human subjects (as defined under 45 CFR 46.102(e))”.

## Supplementary Information

Supplementary Information

## References

[1] Halonen SK, Weiss LM (2013). Toxoplasmosis. Handb. Clin. Neurol..

[2] Flegr J, Prandota J, Sovickova M, Israili ZH (2014). Toxoplasmosis—a global threat. Correlation of latent toxoplasmosis with specific disease burden in a set of 88 countries. PLoS ONE.

[3] Blader IJ, Coleman BI, Chen CT, Gubbels MJ (2015). Lytic cycle of *Toxoplasma gondii*: 15 years later. Annu. Rev. Microbiol..

[4] Montoya JG, Liesenfeld O (2004). Toxoplasmosis. Lancet.

[5] Mendez OA, Koshy AA (2017). Toxoplasma gondii: Entry, association, and physiological influence on the central nervous system. PLoS Pathog..

[6] Dunay IR, Gajurel K, Dhakal R, Liesenfeld O, Montoya JG (2018). Treatment of toxoplasmosis: Historical perspective, animal models, and current clinical practice. Clin. Microbiol. Rev..

[7] Ben-Harari RR, Goodwin E, Casoy J (2017). Adverse event profile of pyrimethamine-based therapy in toxoplasmosis: A systematic review. Drugs R D.

[8] Konstantinovic N, Guegan H, Stajner T, Belaz S, Robert-Gangneux F (2019). Treatment of toxoplasmosis: Current options and future perspectives. Food Waterborne Parasitol..

[9] Iaccheri B (2008). Adverse drug reactions to treatments for ocular toxoplasmosis: A retrospective chart review. Clin. Ther..

[10] Guaraldo L (2018). Ocular toxoplasmosis: Adverse reactions to treatment in a Brazilian cohort. Trans. R. Soc. Trop. Med. Hyg..

[11] Sakaeda T, Tamon A, Kadoyama K, Okuno Y (2013). Data mining of the public version of the FDA adverse event reporting system. Int. J. Med. Sci..

[12] Ahmad SR (2003). Adverse drug event monitoring at the food and drug administration. J. Gen. Intern. Med..

[13] Banda JM (2016). A curated and standardized adverse drug event resource to accelerate drug safety research. Sci Data.

[14] Alday PH, Doggett JS (2017). Drugs in development for toxoplasmosis: Advances, challenges, and current status. Drug Des. Dev. Ther..

[15] Chedid V, Vijayvargiya P, Camilleri M (2018). Advantages and limitations of the federal adverse events reporting system in assessing adverse event reporting for eluxadoline. Clin. Gastroenterol. Hepatol..

[16] Rajapakse S, Chrishan Shivanthan M, Samaranayake N, Rodrigo C, Deepika FS (2013). Antibiotics for human toxoplasmosis: a systematic review of randomized trials. Pathog. Glob. Health.

[17] Ho JM, Juurlink DN (2011). Considerations when prescribing trimethoprim-sulfamethoxazole. CMAJ.

[18] Fukuda A (2017). Comparison of the adverse event profiles of conventional and liposomal formulations of doxorubicin using the FDA adverse event reporting system. PLoS ONE.

[19] Caumes E (1995). Adverse cutaneous reactions to pyrimethamine/sulfadiazine and pyrimethamine/clindamycin in patients with AIDS and toxoplasmic encephalitis. Clin. Infect. Dis..

[20] Bosch-Driessen LH (2002). A prospective, randomized trial of pyrimethamine and azithromycin vs pyrimethamine and sulfadiazine for the treatment of ocular toxoplasmosis. Am. J. Ophthalmol..

[21] Furtado JM, Smith JR, Belfort R, Gattey D, Winthrop KL (2011). Toxoplasmosis: A global threat. J. Glob. Infect. Dis..

[22] Torgerson PR, Mastroiacovo P (2013). The global burden of congenital toxoplasmosis: A systematic review. Bull. World Health Organ.

[23] Welker Y (1993). Interaction between human immunodeficiency virus and *Toxoplasma gondii* replication in dually infected monocytoid cells. Infect. Immun..

[24] Alfonzo M (2002). Temporary restoration of immune response against *Toxoplasma gondii* in HIV-infected individuals after HAART, as studied in the hu-PBMC-SCID mouse model. Clin. Exp. Immunol..

[25] Ngo HM (2017). Toxoplasma modulates signature pathways of human epilepsy neurodegeneration & cancer. Sci. Rep..

[26] Derouin F, Santillana-Hayat M (2000). Anti-toxoplasma activities of antiretroviral drugs and interactions with pyrimethamine and sulfadiazine in vitro. Antimicrob. Agents Chemother..

[27] Neville AJ (2015). Clinically available medicines demonstrating anti-toxoplasma activity. Antimicrob. Agents Chemother..

[28] Wong CK, Ho SS, Saini B, Hibbs DE, Fois RA (2015). Standardisation of the FAERS database: A systematic approach to manually recoding drug name variants. Pharmacoepidemiol. Drug Saf..

[29] Rothman KJ, Lanes S, Sacks ST (2004). The reporting odds ratio and its advantages over the proportional reporting ratio. Pharmacoepidemiol. Drug Saf..

[30] Pittman KJ, Knoll LJ (2015). Long-term relationships: The complicated interplay between the host and the developmental stages of *Toxoplasma gondii* during acute and chronic infections. Microbiol. Mol. Biol. Rev..

[31] Lopez-Gonzalez E, Herdeiro MT, Figueiras A (2009). Determinants of under-reporting of adverse drug reactions: A systematic review. Drug Saf..

[32] Hoffman KB, Dimbil M, Erdman CB, Tatonetti NP, Overstreet BM (2014). The weber effect and the united states food and drug administration's adverse event reporting system (FAERS): Analysis of sixty-two drugs approved from 2006 to 2010. Drug Saf..

[33] Yoshimura K (2013). A survey of the FAERS database concerning the adverse event profiles of alpha1-adrenoreceptor blockers for lower urinary tract symptoms. Int. J. Med. Sci..

