# Supplementary material for: Adverse outcomes associated with the treatment of Toxoplasma infections
Source: Sci Rep. 2021 Jan 13;11:1035. doi: 10.1038/s41598-020-80569-7 (PMC7806722; doi:10.1038/s41598-020-80569-7)
Supplement: Supplementary file 1 — Supplementary Information [file 41598_2020_80569_MOESM1_ESM.pdf]

Adverse outcomes associated with the treatment of *Toxoplasma* infections

Ahmed M. Shammaa[1], Thomas G. Powell[1], Imaan Benmerzouga, Ph.D.[1*]

[1] Department of Biomedical Sciences, West Virginia School of Osteopathic Medicine, Lewisburg, WV

*** Corresponding author**
ibenmerzouga@osteo.wvsom.edu, Phone 304-647-6214

Figure S1

A.

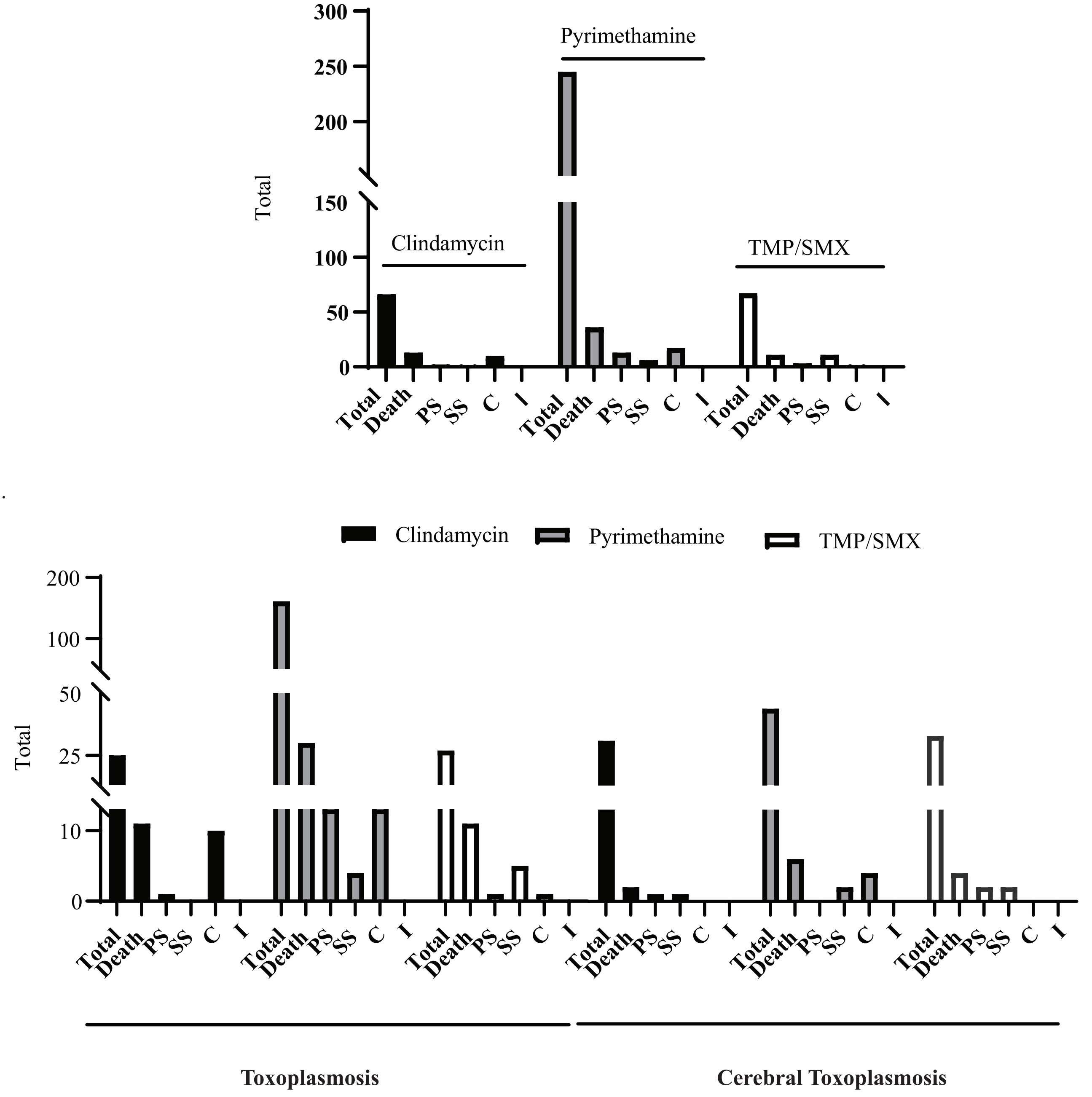

Figure S1: Analysis of reports containing clindamycin, pyrimethamine or TMP/SMX in the treatment of *Toxoplasma* infections in FAERS database. A. Role of clindamycin, pyrimethamine or TMP/SMX in the adverse outcome of death across the indications of *Toxoplasma* infection. B. Role of clindamycin, pyrimethamine or TMP/SMX in the adverse outcome of death based on manifestation of *Toxoplasma* infection in FAERS database.

Figure S2

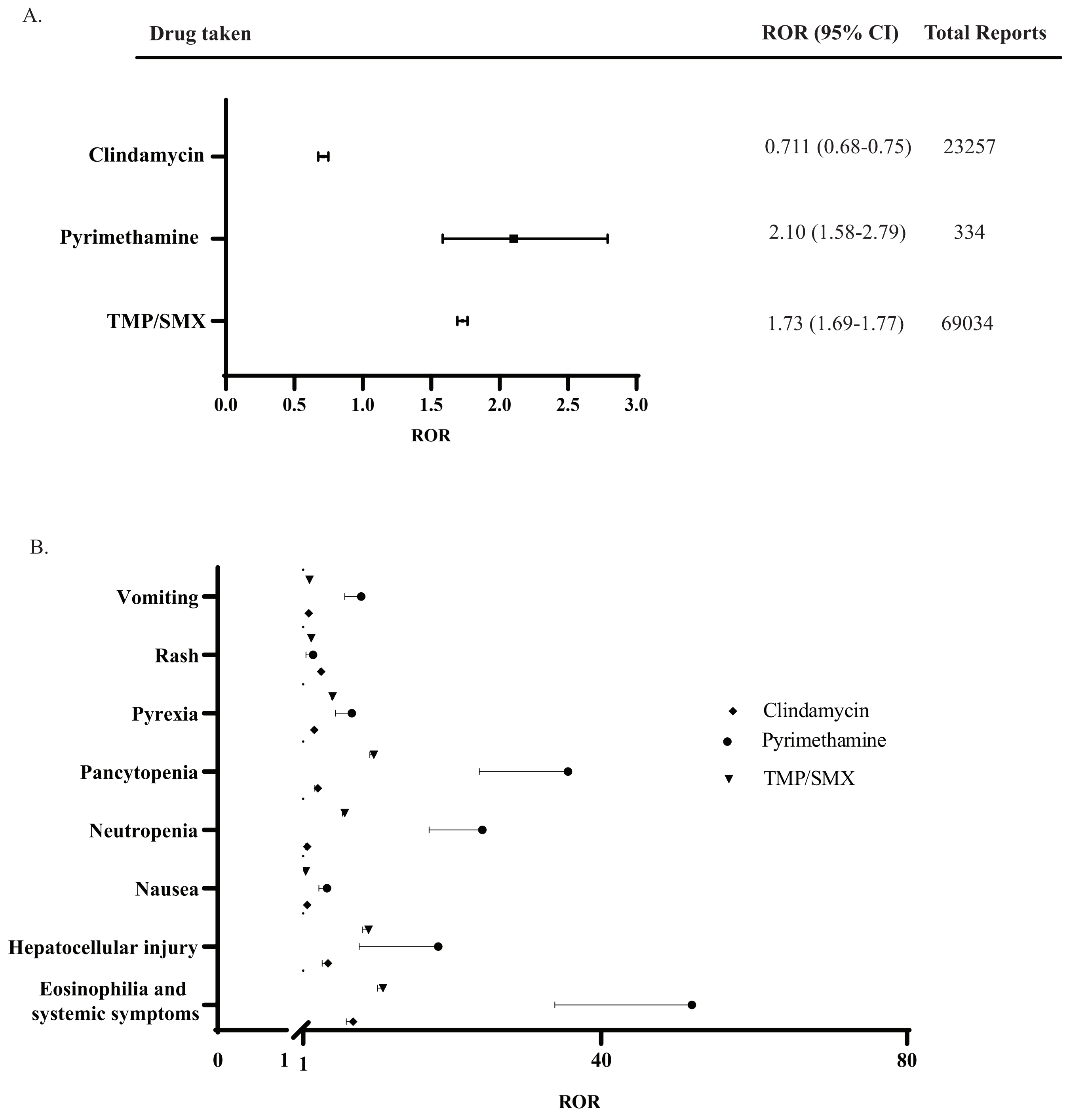

Figure S2: Reporting odds ratio (ROR) of the adverse outcome of death and adverse reactions for clindamycin, pyrimethamine or TMP/SMX in all indications other than *Toxoplasma* infections in FAERS database. A. ROR of the adverse outcome of death for clindamycin, pyrimethamine or TMP/SMX. B. ROR of the adverse events for clindamycin, pyrimethamine or TMP/SMX. Error bars represent the lower limit of the 95% CI.

Table S1

Year	Cerebral Toxo	Ocular Toxo	Congetial Toxo	Toxoplasmosis	Total cases	Total per year
	183	57	5	258	503	
2013	15	5	0	13		33
2014	24	5	2	14		45
2015	22	7	0	15		44
2016	11	5	1	48		65
2017	21	9	1	48		79
2018	33	12	1	72		118
2019	57	14	0	48		119

Table S1: Total cases of *Toxoplasm*a infection manifestation in FAERS database from 2013-2019 after de-duplication (see methods for details)

Table S2

Adverse outcome	FAERS code	FAERS meaning text
Death (DE)	DE	Death
Serious (SE)	LT	Life threatening
	HO	Hospitalization
	OT	Other Serious (Important Medical Event)
	DS	Disability
	CA	Congenital Anomaly
	RI	Required intervention to prevent permanent Impairment/Damage

Note: For *Toxoplasma* infections cases, CA or RI were not listed.

Table S2: FAERS code/meaning in adverse outcome file. SE was assigned to cases with any adverse outcome code excluding death (DE). Death (DE) was assigned to cases that included the adverse outcome of death.

Table S3

Drug name OR group	FAERS drug names AND spelling
Albuterol	ALBUTEROL
Antifungals	AMBISOME, FLUCONAZOLE. TRIFLUCAN, Voriconazole (Unknown)
Antivirals	Aciclovir, COBICISTAT/ELVITEGRAVIR/EMTRICITABINE/TENOFOVIR ALAFENAMIDE FUMARATE, EFAVIRENZ, EFAVIRENZ/EMTRICITABINE/TENOFOVIR DISOPROXIL FUMARATE, EMTRICITABINE W/TENOFOVIR, Emtricitamine, Tenofovir, FAMVIR, LAMIVUDINE, Lopinavir/Ritonavir, PREZISTA, RALTEGRAVIR, STAVUDINE, ZIDOVUDINE
Atovaquone	ATOVAQUONE, MEPRON, SAMTIREL, WELLVONE, Wellvone
Azithromycin	AZITHROMYCIN, AZITHROMYCIN ANHYDROUS, ZITHROMAC, ZITHROMAX, Zithromax
Ciprofloxacin	CIPRINOL
Clindamycin	CLIDAMACIN, CLINDAMYCIN, CLINDAMYCIN (CLINDAMYCIN), CLINDAMYCIN HCL, CLINDAMYCIN HYDROCHLORIDE, CLINDAMYCINE /00166002/, DALACINE, DALACINE FORMES INJECTABLES, Dalacin, Dalacine
Clonazepam	CLONAZEPAM
Corticosteroids	CORTICOSTEROIDS, DEXAFREE, DEXAMETHASON /00016001/, DEXAMETHASONE, DUREZOL, ENCORTON, OZURDEX, PREDNISOLONE (PREDNISOLONE), PREDNISOLONE, PREDNISONE,
Cyclosporine	CYCLOSPORINE (NON-MODIFIED) 25MG APOTEX
Dalteparin	FRAGMIN
Folic acid/Folate	FOLIC ACID, Foliamin,
Folinic acid	CALCIUM FOLINATE, FOLINIC ACID, Folinic acid, LEDERFOLIN, LEUCOVORIN, LEUCOVORIN (CALCIUM FOLINATE), LEUCOVORIN /00566701/, LEUCOVORIN CALCIUM, LEUCOVORIN /00566701/, LEUCOVORIN CALCIUM (NON-SPECIFIC), LEUCOVORINE, LEUKOVORIN, Lederfolin, Lederfoline
Gabapentin	GABAPENTIN
Gadolinium	GADOLINIUM (UNSPECIFIED)
Glaucoma medications	BRINZOLAMIDE, CARTEOLOL, COSOPT, TIMOLOL.
Haloperidol	HALOPERIDOL.
Levetiracetam	KEPPRA, Keppra, LEVETIRACETAM MYLAN PHARMA 250 mg, comprim? pellicul?, LEVETIRACETAM.
Levothyroxine	LEVOTHYROXINE
Meropenem	MEROPENEM.
Metronidazole	FLAGYL, FLAGYL /00012501/
Minocycline	MINOCYCLINE
NSAIDs	INDOCOLLYRE, KETOROLAC, ACETYLSALICYLIC ACID
Ofloxacin	OFLOXACIN.

Table S3-continued

Drug name OR group	FAERS drug names AND spelling
Pyrimethamine	DARAPRIM, PIRIMETAMINA, PIRIMETHAMINE, PYRIMETHAMINE, PYRIMETHAMINE/PYRIMETHAMINE BITARTRATE, Pyrimethamine, pyrimethamine
Pyrimethamine (+) Sulfonamides	MALOCIDE, MALOCIDE /00112501/, MALOCIDE (PYRIMETHAMINE), MALOCIDE (PYRIMETHAMINE\SULFADOXINE), MALOCIDE /00112501/, MALOCIDE 50 mg, comprim?, , MALOCIDE [PYRIMETHAMINE], METAKELFIN, Malocide, PYRIMETHAMINE (+) SULFADOXINE, PYRIMETHAMINE W/SULFADIAZINE, PYRIMETHAMINE\SULFALENE, Pyrimethamine/Sulfadoxine, SULFADOXINE PYRIME, SULFADOXINE PYRIMETHAMINE, Sulfadoxine pyrimethamine, malocide, pyrimethamine (+) sulfadoxine
Ranibizumab	LUCENTIS
Spiramycin	ROVAMICINA, ROVAMYCINE, Rovamycin, SPIRAMYCIN, Spiramycin
Sulfonamides	ADIAZINE, ADIAZINE (ADIAZINE), ADIAZINE 500 mg, comprim?, Adiazine, SILVER SULFADIAZENE, SULFADIAZIN, SULFADIAZINA, SULFADIAZINE, SULFADIAZINE (SANDOZ), SULFADIAZINE TABLETS, USP, SULFADIAZINE/SULFADIAZINE SILVER/SULFADIAZINE SODIUM, SULFADOXINE, SULFASALAZINE, SULPHADIAZINE, SULPHADIAZINE /00076401/, SULPHADOXINE, Salazopyrin, Sulfadiazin, adiazine
Tesamorelin	EGRIFTA
Trimethoprim (+) Sulfamethoxazole	BACTRAMIN, BACTRIM, BACTRIM DS, BAKTAR, Baktar, CO-TRIMOXAZOLE, COTRIM, COTRIMOXAZOLE, SULFAMETHIZOLE/TRIMETHOPRIM, SULFAMETHOXAZOLE AND TRIMETHOPRIM, SULFAMETHOXAZOLE/TRIMETHOPRIM, SULPHAMETHOXAZOLE/TRIMETHOPRIM, Septrin, Sulfamethoxazole/trimethoprim, Sulphamethoxazole/Trimethoprim, Trimethoprim + sulfamethoxazole, Trimethoprim+Sulfamethoxazole, Trimethoprim-Sulfamethoxazole (Unknown), sulfamethoxazole (+) trimethoprim, sulfamethoxazole/trimethoprim
Unknown drug	UNKNOWN DRUG

Table S3: Mapping of anti-*Toxoplasma* agents from FAERS DRUG file (see methods for details)

Table S4

Drug name OR group with < 10 adverse outcome reports
Albuterol
Antifungals
Ciprofloxacin
Clonazepam
Cyclosporine
Dalteparin
Folic acid/Folate
Gabapentin
Gadolinium
Glaucoma medications
Haloperidol
Levetiracetam
Levothyroxine
Meropenem
Metronidazole
Minocycline
NSAIDs
Ofloxacin
Ranibizumab
Tesamorelin
Unknown drug

Table S4: List of drugs with < 10 adverse outcome reports
